# Electrospun Polytetrafluoroethylene Nanofibrous Membrane for High-Performance Self-Powered Sensors

**DOI:** 10.1186/s11671-019-3091-y

**Published:** 2019-07-25

**Authors:** Shizhe Lin, Yongliang Cheng, Xiwei Mo, Shuwen Chen, Zisheng Xu, Bingpu Zhou, He Zhou, Bin Hu, Jun Zhou

**Affiliations:** 1 Wuhan National Laboratory for Optoelectronics, Huazhong University of Science and Technology, Wuhan, 430074 China; 2 Key Laboratory of Synthetic and Natural Functional Molecule, Chemistry of the Ministry of Education, College of Chemistry and Materials Science, Northwest University, Xi’an, 710069 China; 3 Institute of Applied Physics and Materials Engineering, University of Macau, Taipa, Macau China

**Keywords:** Electrospinning, Polytetrafluoroethylene, Nanofiber, Nanogenerator, Self-powered sensor, Wearable electronic

## Abstract

Polytetrafluoroethylene (PTFE) is a fascinating electret material widely used for energy harvesting and sensing, and an enhancement in the performance could be expected by reducing its size into nanoscale because of a higher surface charge density attained. Hence, the present study demonstrates the use of nanofibrous PTFE for high-performance self-powered wearable sensors. The nanofibrous PTFE is fabricated by electrospinning with a suspension of PTFE particles in dilute polyethylene oxide (PEO) aqueous solution, followed by a thermal treatment at 350 °C to remove the PEO component from the electrospun PTFE-PEO nanofibers. The obtained PTFE nanofibrous membrane exhibits good air permeability with pressure drop comparable to face masks, excellent mechanical property with tensile strength of 3.8 MPa, and stable surface potential of − 270 V. By simply sandwiching the PTFE nanofibrous membrane into two pieces of conducting carbon clothes, a breathable, flexible, and high-performance nanogenerator (NG) device with a peak power of 56.25 μW is constructed. Remarkably, this NG device can be directly used as a wearable self-powered sensor for detecting body motion and physiological signals. Small elbow joint bending of 30°, the rhythm of respiration, and typical cardiac cycle are clearly recorded by the output waveform of the NG device. This study demonstrates the use of electrospun PTFE nanofibrous membrane for the construction of high-performance self-powered wearable sensors.

**Electronic supplementary material:**

The online version of this article (10.1186/s11671-019-3091-y) contains supplementary material, which is available to authorized users.

## Introduction

Wearable electronics have been considered as an important class of the next-generation electronics because of their wide applications in a lot of fields such as health monitoring, artificial skin, and human-interactive interfaces [1, 2]. The booming development of wearable electronics has propelled a huge demand of wearable sensors as basic functional parts of those electronics [3]. Great opportunities are thus posed in the development of wearable sensors that are lightweight, flexible, stretchable, and can be conformally in contact with particular surfaces. To attain these capabilities, novel functional materials and approaches in material processing at nanoscale are required for the construction of sensor devices [4–6].

As one kind of those most used wearable sensors, flexible pressure sensors which can effectively convert mechanical force into electrical signal have wide application for body motion detection [7] and health monitoring [8, 9]. Recently, many groups have contributed to the advancement in highly sensitive and flexible pressure sensors based on piezoresistivity [10] and capacitance mechanism [11, 12]. However, these devices are mainly powered by an external energy source, which makes them complicated and expensive, greatly limiting their application. It is necessary to integrate a self-powered system into the device to dismiss the external power supply unit. Fortunately, there is sufficient energy generated from human’s daily activities such as arm motion, body heat, and breathing [13], which could be used for powering the sensors. Thus, several types of nanogenerators (NGs) based on piezoelectric effect [14], triboelectric effect [2], and electrostatic effect [15] have been constructed to effectively utilize human body energy as a power source for self-powered sensors.

Polytetrafluoroethylene (PTFE), as an important member of both the triboelectric and electret families, has been widely used for energy harvesting and sensor devices [16–18]. Owing to its helical chain conformation with a uniform coverage of fluorine atoms on carbon backbone, PTFE shows good flexibility, ultrahigh chemical inertness, and excellent thermal stability. These characteristics make PTFE a fascinating material for a lot of applications but also cause significant difficulty in its processing. Thus, most of the reports on the utilization of PTFE for energy harvesting and sensing were focused on the use of commercially available PTFE thin-films without any post-treatment [17, 18] or treated films by high-cost processing such as reactive ion etching [19, 20]. It is well known that increasing the microscopic surface area of the triboelectric generator can enhance its effective surface charge density at the same time and therefore promotes its output performance as well [21]. Recently, using electrospun PTFE nanofibrous membrane as an alternative to commercial PTFE thin-film has been proved to be an effective method to promote the performance of triboelectric NG, because of the much larger surface area of the former [22]. The surface charge density is also the key factor that determines the performance of an electret, suggesting electrospun PTFE nanofibrous membrane may be used for the construction of high-performance electret devices.

Herein, we report on the application of electrospun PTFE nanofibrous membrane as a high-performance electret NG for self-powered sensors. The design of this work shows several advantages. First, the self-powered sensor device was assembled by simply sandwiching the electrospun PTFE nanofibrous membrane with two pieces of conductive cloth. This fabrication process is facile, low cost, and easy to scale up. Second, unlike PTFE thin film, the nanofibrous membrane shows good air permeability. Thus, the assembled sensor device is breathable, satisfying the requirement of wearable electronics. Third, the assembled device can efficiently convert mechanical energy into electricity with a high peak power of 56.25 μW and long-time operation stability. At last, as a wearable sensor, the device can sensitively monitor body motion as well as physiological signals including respiration and heartbeat, showing the potential in application for both body motion and health monitoring.

## Methods

### Fabrication of the PTFE Nanofibrous Membrane

The PTFE nanofibrous membrane was fabricated by a two-step method. First, a PTFE-PEO (polyethylene oxide) nanofibrous membrane was fabricated by electrospinning with a Kangshen KH1001 electrospinning machine. To prepare the solution for electrospinning, 18 g PTFE suspension (60 wt%, Aladdin) was added into 6.0 g deionized water forming a uniform suspension, then 0.4 g PEO (*M*_w_ = 5 × 10^6^, Aladdin) was added into the above solution to adjust its viscosity. After magnetic stirring for 48 h, the mixture was loaded in a 5-ml syringe with a stainless steel needle tip. During the electrospinning, a high voltage of 25 kV was applied on the needle tip and the solution was pumped out of the needle at a speed of 1.5 mL h^−1^. The ejected fibers were collected on a rotating metal drum with a rotation speed of 200 rpm for 1 h. The distance between the needle tip and the collector was fixed as 18 cm. Then, the as-prepared PTFE-PEO nanofibrous membrane was subjected to a thermal treatment at 350 °C in ambient atmosphere for 10 min with a heating rate of 2 °C min^−1^ to obtain the PTFE nanofibrous membrane.

### Corona Charging

For corona charging, the PTFE nanofibrous membrane with one side grounded was placed 5 cm below a corona needle, which was connected to a high-voltage source (DW-N503-4ACDE). A voltage of − 20 kV was then applied to the corona needle for 5 min.

### Assembly of the Self-Powered Sensor Device

First, the corona charged PTFE nanofibrous membrane was stored at ambient condition for 1 day because of the sharp decay of its surface potential just after corona charging. Then, it was fixed between two 250-μm-thick polyethylene terephthalate spacers. Subsequently, the PTFE nanofibrous membrane was sandwiched into two conductive cloth electrodes to form the sensor device with an effective size of 4 × 4 cm^2^.

### Characterization

The morphology, composition, and crystallinity of the samples were characterized by field emission scanning electron microscopy (FE-SEM, NANOSEM 450, FEI), X-ray photoelectron spectroscopy (XPS, ESCALab250, Thermo Scientific), Fourier-transform infrared spectroscopy (FTIR, Vertex 70, Bruker), and X-ray diffraction (XRD, X’ Pert Pro MPD, PANalytical B.V.), respectively. The surface potential, mechanical property, and pressure drop of the membrane were detected by an electrometer (EST102, Huajing Beijing, China), a universal testing machine (REGER RW-T10), and a pressure transmitter (DP102, Sike instruments), respectively. The output current of the sensor device was measured by a Stanford low-noise current preamplifier (Model SR570 and NI PCI-6259). Besides testing the output performance of the device with different loading resistances, all the other measurements were conducted in short circuit condition.

## Results and Discussion

**Fig. 1:**
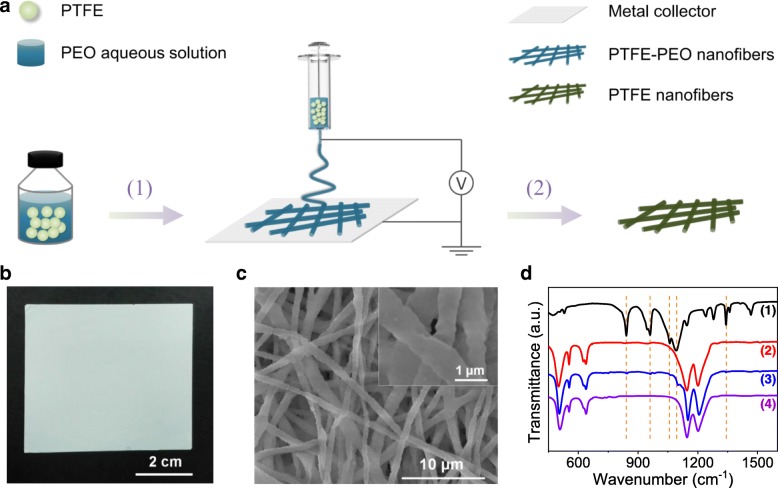
**a** Schematic diagram showing the two-step fabrication of PTFE nanofibrous membrane: (1) electrospinning to obtain PTFE-PEO nanofibrous membrane and (2) thermal treatment to remove PEO from the electrospun PTFE-PEO nanofibrous membrane. **b** Digital photograph and **c** SEM image of the PTFE nanofibrous membrane with the inset showing a magnified view. **d** FTIR spectra of the (1) pristine PEO, (2) pristine PTFE, (3) electrospun PTFE-PEO nanofibrous membrane, and the (4) PTFE nanofibrous membrane, with the dashed orange lines indicate the main peaks of PEO

The PTFE nanofibrous membrane was fabricated by a two-step approach, as schematically shown in Fig. 1a. Because of the outstanding chemical resistance of PTFE, it cannot be dissolved in any solvents, so it is difficult to directly electrospin PTFE solution into nanofibers. To overcome this issue, a two-step approach was generally used for the fabrication of PTFE nanofibers [23, 24]. First, a nanofibrous PTFE composite was prepared by electrospinning, using a water-soluble polymer as a carrier for the dispersion of PTFE particles. Then, a post-thermal treatment was applied to remove the carrier to obtain PTFE nanofibers. In this study, PEO was utilized as a carrier because of its good water solubility and low melting point. Using the PTFE particle-suspended PEO aqueous solution as the precursor for electrospinning, PTFE-PEO nanofibers with diameters of 500~800 nm were successfully obtained, as shown in Additional file 1: Figure S1. Because the small amount of PEO (PEO/PTFE = 1/27 in the precursor solution) cannot fully package the PTFE particles, the as-prepared PTFE-PEO nanofibers show rough surface and phase composition of only PTFE (Additional file 1: Figure S1b). In order to obtain pure PTFE nanofibers, a thermal treatment was employed to remove PEO and fused PTFE particles together. According to a previous study, PTFE melts at ~ 327 °C and is thermal stable until ~ 500 °C [24]. Thus, a temperature of 350 °C, slightly higher than the melting temperature of PTFE, was chosen to remove PEO and fuse PTFE nanoparticles together to form continuous nanofibers. As shown in Fig. 1b, PTFE nanofiber web with a size of 5 cm × 5 cm was obtained after calcination. SEM study revealed that the fiber morphology was well maintained after calcination (Fig. 1c). The interconnection of some PTFE nanofibers and disappearance of PTFE nanoparticles on the nanofibers demonstrated the fusion of nanoparticles (inset of Fig. 1c). The elimination of the PEO component from the nanofibers was revealed by FTIR study. As shown in Fig. 1d, the pristine PEO exhibits several prominent peaks at 841, 947, 1059, 1092, and 1342 cm^−1^, corresponding to the vibrations of the CH_2_ and CO groups [22, 25]. On the other hand, there are five strong peaks showed up in the FTIR spectrum of the pristine PTFE, among which the most prominent ones at 1146 and 1201 cm^−1^ are characteristic of CF_2_ symmetric and asymmetric stretching modes, respectively [26], and the peaks at 512, 554, and 639 cm^−1^ could be assigned to the rocking, deformation, and wagging modes of CF_2_, respectively [27]. The peaks assigned to PEO are still observable in the spectrum of the electrospun PTFE-PEO nanofibrous membrane despite the low content of the PEO component (as indicated by the dashed orange lines in Fig. 1d). After sintered at 350 °C, these peaks are completely disappeared, resulting in the bare PTFE composition of the nanofibrous membrane.

**Fig. 2:**
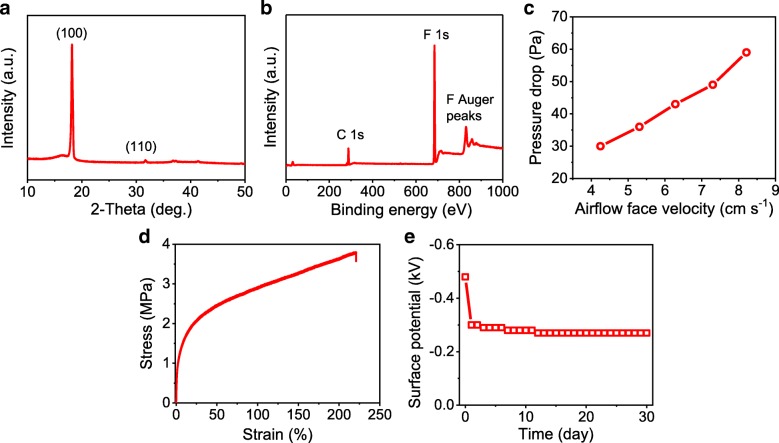
Characterization on the PTFE nanofibrous membrane: **a** XRD pattern, **b** XPS spectrum, **c** pressure drop as a function of gas flow rate, **d** stress−strain curve, and **e** variation of surface potential in 30 days.

Figure 2 shows a set of characterization results on the PTFE nanofibrous membrane. Similar to the precursor PTFE-PEO sample, the PTFE nanofibrous membrane consists of only PTFE phase. As shown in Fig. 2a, there are two diffraction peaks located at 18.2° and 31.7° on the XRD pattern, corresponding to the (100) and (110) planes of PTFE respectively. XPS study further illuminates its composition of bare PTFE. The XPS pattern exhibits characteristic peaks of C 1°s and F 1°s centered at ~ 286 and ~685 eV, respectively (Fig. 2b). While the characteristic peak of O 1 s which generally appears at ~ 532 eV could not be observed [28], suggesting the PEO component has been completely eliminated during the thermal treatment. To evaluate the suitability of using the PTFE nanofibrous membrane as a wearable electret sensor, its properties related to the requirement of this specific application has also been characterized. Figure 2c gives the pressure drops when the air goes across the membrane at various flow rates. The pressure drop keeps almost linear relationship with gas flow rate in the tested extent, and its values are quite small, even comparable to those of filter face masks [29], demonstrating the good air permeability of the membrane. Plausibly due to the interconnection of fiber network, the membrane also exhibits excellent mechanical property with a tensile strength of ~ 3.8 MPa and elongation at break of 220% (Fig. 2d), which satisfies the requirement of wearable electronics. Figure 2e shows the surface potential variation of the membrane within 30 days. The value decays sharply from about − 480 to − 300 V after storing the membrane at ambient condition for 1 day and then decreases slowly in the following 11 days, finally keeps stable at − 270 V. The good air permeability, excellent mechanical property, and stable surface potential of the PTFE nanofibrous membrane reveal its potential application for wearable self-powered sensing.

**Fig. 3:**
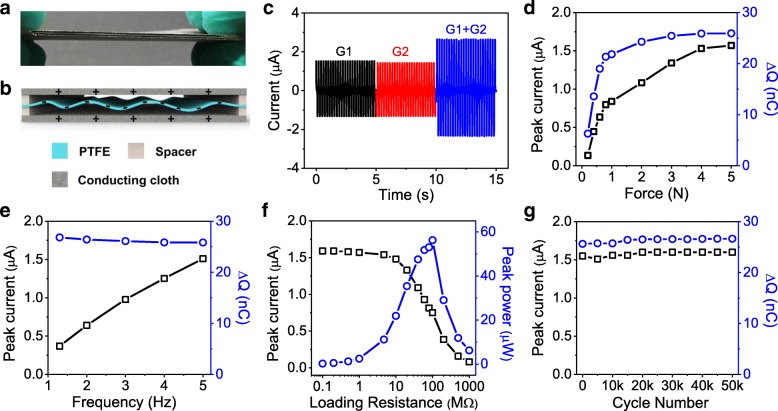
**a** Digital photograph of the NG device and **b** a schematic illustration of its structure. **c** Output current of two individual NG devices (G1 and G2) and a parallel connection of them (G1 + G2). **d** Output of the NG with different stimulation forces at 5 Hz. **e** Output of the NG at different frequencies with a stimulation force of 5 N. **f** Output of the NG with different loading resistance. **g** Cycling stability of the NG

Relied on its charge storage capability, the PTFE nanofibrous membrane could be utilized to fabricate electret NG. In order to keep its air permeability when integrated into a device, commercial conducting cloth with excellent air permeability was employed as an electrode to construct the electret NG (Additional file 1: Figure S2). First, two ends of the PTFE nanofibrous membrane were fixed between two spacers; then, the membrane was sandwiched into two pieces of conducting carbon clothes forming the NG device with an effective size of 4 cm × 4 cm (Fig. 3a). The negative surplus charge in the PTFE nanofibers would induct positive charge in the top and bottom electrodes with a total amount equal to that of the negative charge (Fig. 3b). In a static state, no charge could be transferred due to the equilibrium state of electric potential distribution. When the equilibrium state was broken by pressing and releasing the device, the change of gap between the PTFE membrane and carbon cloth electrodes would lead to a change of the capacitance and thus resulted in a redistribution of the charges between the two electrodes, producing an alternate transient current flowing through the external circuit. The working mechanism of this sandwich structure NG is similar to those reported arch structure NGs [17, 30]. Nevertheless, the NG shown in the present work is much easier to be constructed and more breathable, compared to those thin film-based arch structure NGs and some other fiber-based NGs [17, 30–34].

As shown in Fig. 3c, the NG exhibited a peak current of ~ 1.5 μA under a stimulation force of 5 N and a frequency of 5 Hz. When two NGs were connected in parallel with the same polarity, the total output current was almost the added value of each one, indicating that the electrical output of the NGs satisfied the linear superposition criterion in the basic circuit connections [35]. The performance of the NG was further systematically studied under different forces and frequencies. At a given frequency, both the peak current and the integral amount of transferred charge (Δ*Q*) increased as an increase of the stimulation force from 1 to 5 N (Fig. 3d and Additional file 1: Figure S3a). A further increase of the stimulation force could not further promote the output because Δ*Q* was only dictated by the amplitude of gap change between PTFE membrane and the electrodes [17], which had already reached the maximal value at a sufficient force of 5 N. Also, due to the capacitance variation mechanism, Δ*Q* kept an almost constant value of ~ 26.9 nC with a variation of frequency because the amplitude of gap change was independent of frequency (Fig. 3e). Nevertheless, the output current increased with the increase of frequency at a given stimulation force (Additional file 1: Figure S3b), because the same amount of charge was transferred in a shorter time. In order to obtain the maximal peak power, the output performance with different external loading resistances was studied at a frequency of 5 Hz and stimulation force of 5 N. As shown in Fig. 3f, the output current kept almost unchanged with a loading resistance of 0.1~10 MΩ and then decreased from ~ 1.5 to 0.081 μA with a further increase of the loading resistance to 1000 MΩ, implying an internal resistance of the NG device between 10 and 1000 MΩ. Based on the definition of power, *P* = *I*^2^*R*, a maximal peak power as high as 56.25 μW could be obtained with a loading resistance of 100 MΩ. Accordingly, the internal resistance of the NG device was deduced to be ~ 100 MΩ, because the maximal power of an NG appears on condition that its internal resistance matches the loading resistance [21]. At last, the cycling stability of the NG was evaluated at a force of 5 N and frequency of 5 Hz. As depicted in Fig. 3g, no obvious deterioration in output current as well as integral amount of transferred charge was found during 50 k cycles, revealing excellent cycling stability of the NG.

**Fig. 4:**
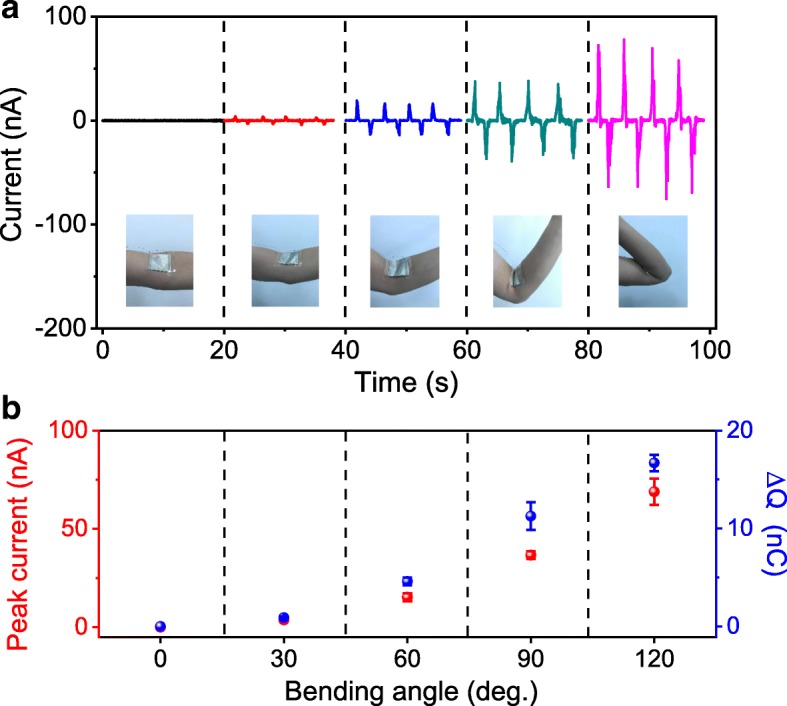
**a** Output signals of the NG at various bending angles of the elbow joint and **b** the corresponding plots of peak current and integral transferred charge

To demonstrate the potential of using the NG as a self-powered sensor for body motion monitoring, the device was fixed over the straightened elbow joint to monitor elbow joint motion. Figure 4a shows the output electrical signals when bending the elbow joint to a series of angles. The current pulses are clearly identifiable even with a small motion of 30° bending and become more and more prominent at elevated bending angles. Figure 4b draws the relationship between the output of the NG and blending angle of the elbow joint. Due to the complicated deformation of the device, the change of gap between the PTFE membrane and carbon cloth electrodes could not be quantitatively correlated to the bending angle of the elbow joint. Thus, the relationship between the output of the NG device and the bending angle of the elbow joint can be only mathematically established but not physically. Nevertheless, the dependence of current and transferred charge on blending angle can effectively denote the state of elbow joint motion, demonstrating the potential application of the NG as a self-powered sensor for real-time monitoring body motion.

**Fig. 5:**
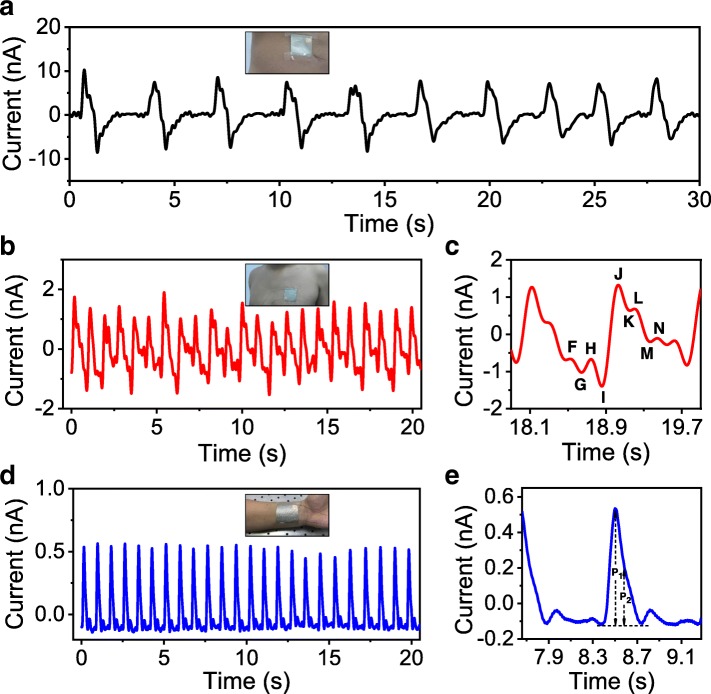
Output signal of the NG attached on different positions of a male’s body: **a** on the abdomen, **b** on the chest, and **d** on the wrist; **c** and **e** are enlarged views of the signal in **b** and **d** respectively

Besides the application for body motion monitoring, the NG can also serve as a self-powered sensor for monitoring physiological signals via attaching the device on specific positions of human body. For instance, when fixing the NG device on the abdomen, the shrinking and expansion of the abdomen during respiration will stimulate the device, generating electrical signals that provide information on respiration. As shown in Fig. 5a, clear alternate current waves with a peak value of 6~10 nA have been recorded, which well match the respiratory rhythm of a male adult with a frequency of ~ 20 times per minute. The NG device can also be used for heartbeat monitoring when fixed on the chest or wrist. The regular pulsation of the heart or artery will stimulate the NG device to produce corresponding periodic current signals as traces of heartbeat. This is the so-called ballistocardiography method, which mechanism is based on tracking subtle mechanical motions generated by the ejection of blood during cardiac cycle [36]. Figure 5b presents the output of the NG device attached on the chest of a male, from which 23 prominent current peaks in 20 s can be unambiguously identified, suggesting a heartbeat rate of ~ 69 beats per minute. This value is in the range of normal extent for a healthy young man (60~100 beats per minute [37]). Furthermore, the signal is capable of comprehensive interpretation to extract information on the detail of each cardiac cycle, which is useful for auxiliary cardiovascular diagnosis [36, 38]. As exampled in Fig. 5c, the electric waveform explicitly tracks the three processes of a typical cardiac cycle, naming presystole (F–G–H), systole (I–J–K), and diastolic (L–M–N) stages [37]. In comparison to the measurement of aortic pulse wave near the heart, monitoring the peripheral arterial pulse by fixing the NG device on the trunk is more convenient. Figure 5d shows the recorded current signal of the NG fixed on the wrist. The sharp current pulses on the pattern clearly record the rhythm of radial artery beating with a frequency of ~ 72 times per minute. Figure 5e is an enlarged view of the waveform, from which two main peaks could be distinguished: the incident blood flow peak *P*_1_ and the reflected peak *P*_2_ from the hand region [37]. Based on the amplitude of these peaks, the radial artery augmentation index (AI_x_ = *P*_2_/*P*_1_), as an important indicator of cardiovascular diseases and target organ damage, could be calculated [39]. According to the acquired data, a statistic value of ~ 54% was obtained, suggesting a normal cardiovascular condition for a 33 years old male.

## Conclusions

In summary, the present work justified the suitability of using electrospun PTFE nanofibrous membrane for the construction of high-performance self-powered wearable sensors. PTFE nanofibrous membrane was successfully fabricated by electrospinning with a PTFE-PEO aqueous suspension and a post-thermal treatment to eliminate the PEO component. Owing to its good air permeability and excellent mechanical and electret properties, the fabricated NG device based on the electrospun PFTE nanofibrous membrane could effectivity convert mechanical energy into electricity with a high peak power of 56.25 μW and long-term cycling stability, showing the potential to be used as a sensitive self-powered wearable sensor. Indeed, the NG was demonstrated to be an excellent wearable sensor that could quantitatively monitor body motion and biological signals including respiration and heartbeat, implying its potential application in wearable electronics for body motion and health monitoring.

## Additional File

Additional file 1: **Figure S1**. a. SEM images and b. XRD pattern of the PTFE-PEO nanofibrous membrane. **Figure S2**. a. SEM image and b. pressure drop as a function of gas flow rate of the conducting carbon cloth. **Figure S3**. a. Output of the NG device with different pressing forces at a frequency of 5 Hz. b. Output of the NG device at different frequencies with a pressing force of 5 N. (PDF 561 kb)

## Data Availability

All data generated in this study are included in the article and its additional file.

## Abbreviations

FE-SEM: Field emission scanning electron microscopy
FTIR: Fourier-transform infrared spectroscopy
NG: Nanogenerator
PEO: Polyethylene oxide
PTFE: Polytetrafluoroethylene
XPS: X-ray photoelectron spectroscopy
XRD: X-ray diffraction
