# Supplementary material for: Electrospun Polytetrafluoroethylene Nanofibrous Membrane for High-Performance Self-Powered Sensors
Source: Nanoscale Res Lett. 2019 Jul 25;14:251. doi: 10.1186/s11671-019-3091-y (PMC6658626; doi:10.1186/s11671-019-3091-y)
Supplement: Supplementary file 1 — Figure S1. a. SEM images and b. XRD pattern of the PTFE-PEO nanofibrous membrane. Figure S2. a. SEM image and b. pressure drop as a function of gas flow rate of the conducting carbon cloth. Figure S3. a. Output of the NG device with different pressing forces at a frequency of 5 Hz. b. Output of the NG device at different frequencies with a pressing force of 5 N. (PDF 561 kb) [file 11671_2019_3091_MOESM1_ESM.pdf]

Additional file 1

Electrospun Polytetrafluoroethylene Nanofibrous Membrane for High-Performance Self-Powered Sensors

Shizhe Lin[1†], Yongliang Cheng[2†], Xiwei Mo[1], Shuwen Chen[1], Zisheng Xu[1], Bingpu Zhou[3], He Zhou[1*], Bin Hu[1] and Jun Zhou[1]

[1]Wuhan National Laboratory for Optoelectronics, Huazhong University of Science and Technology, Wuhan 430074, China

[2]Key Laboratory of Synthetic and Natural Functional Molecule, Chemistry of the Ministry of Education, College of Chemistry and Materials Science, Northwest University, Xi'an 710069, China

[3]Institute of Applied Physics and Materials Engineering, University of Macau, Taipa, Macau, China

[†]Shizhe Lin and Yongliang Cheng contributed equally to this work.

[*]Correspondence: zhouhehust@gmail.com

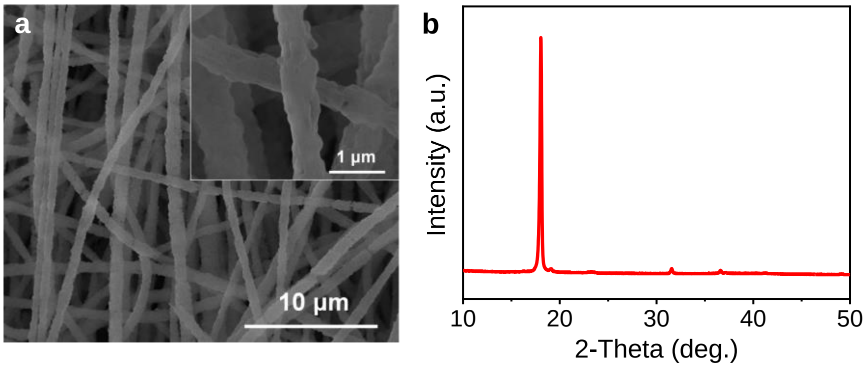

Fig. S1 a SEM images and **b** XRD pattern of the PTFE-PEO nanofibrous membrane.

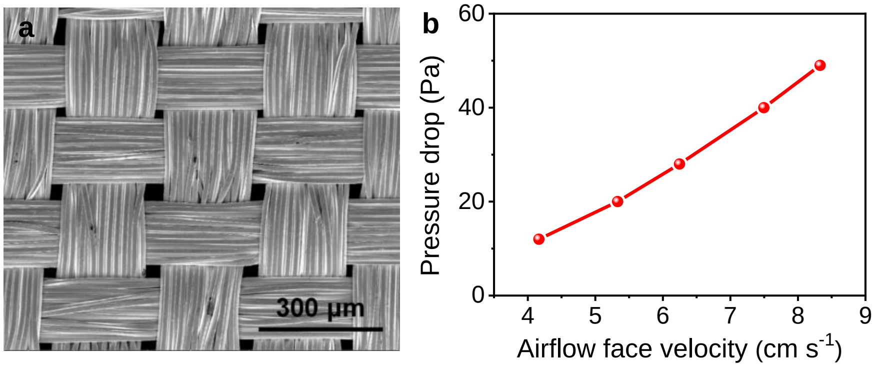

Fig. S2 a SEM image and **b** pressure drop as a function of gas flow rate of the conducting carbon cloth.

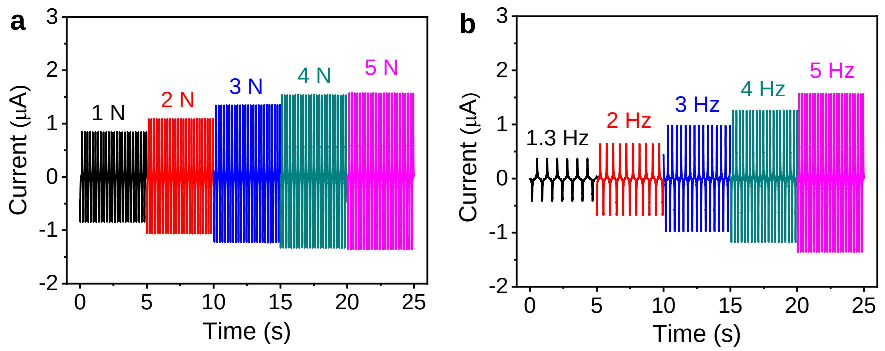

Fig. S3 a Output of the NG device with different pressing forces at a frequency of 5 Hz. **b** Output of the NG device at different frequencies with a pressing force of 5 N.
